# Extractive Electrospray Ionization Mass Spectrometry for Rapid Analysis of Organic and Inorganic Selenium in Honey

**DOI:** 10.3390/molecules30214206

**Published:** 2025-10-28

**Authors:** Xinrui Zhang, Yuqi Qiu, Huiyu Xing, Feixiang Yang, Peng Zeng, Hao Fan, Huanwen Chen, Xiaowei Fang

**Affiliations:** 1 The Jiangxi Province Key Laboratory for Diagnosis, Treatment, and Rehabilitation of Cancer in Chinese Medicine, Jiangxi University of Chinese Medicine, Nanchang 330004, China; zxr030122@outlook.com (X.Z.); qyq1205@outlook.com (Y.Q.); xinghuiyu1@jutcm.edu.cn (H.X.); yangfeixiang2024@163.com (F.Y.); zpctcj@outlook.com (P.Z.); chw8868@gmail.com (H.C.); 2 Department of Pharmacy, Jiangxi University of Chinese Medicine, Nanchang 330004, China; fanhao11@aliyun.com; 3 State Key Laboratory for Quality Ensurance and Sustainable Use of Dao-di Herbs, Beijing 100700, China

**Keywords:** EESI-MS, honey, selenium, rapid analysis

## Abstract

Selenium, a crucial trace element for human health, plays a vital role in maintaining well-being. Its insufficiency can cause various diseases, highlighting the need for adequate selenium intake in daily diets. Honey, containing diverse selenium compounds, serves as a beneficial selenium supplement. By leveraging the distinctive physicochemical properties of honey, we employed reactive extractive electrospray ionization mass spectrometry (EESI-MS) to rapidly analyze the presence of both organic selenium (selenomethionine) and inorganic selenium (sodium selenite) in diluted honey samples. We successfully identified selenomethionine (SeMet) and sodium selenite. Calibration curves constructed for SeMet and sodium selenite demonstrated excellent linear relationships within the concentration range of 0.5 to 50 µg/L. The limits of detection (LOD) for SeMet and sodium selenite were determined to be 2.94 µg/kg and 5.18 µg/kg, respectively, while the limits of quantification (LOQ) were 9.52 µg/kg and 17.4 µg/kg, respectively. Furthermore, spiked recoveries ranged from 90.6% to 105%. The average analysis time is 2 min. This study presents a precise, rapid, and convenient method for selenium determination in diluted honey. Given the limited sample size in this preliminary study, future research with larger cohorts is required to validate our findings.

## 1. Introduction

Selenium is deemed an indispensable trace element that has significant impacts on human health [1]. Its intake pathways are diverse in daily life, including pharmaceutical supplements and dietary consumption, with the latter predominating as the principal route of ingestion [2]. Selenium exists in two forms: organic and inorganic. As elucidated in the study [3] on selenoproteins in health, organic selenium (such as selenoproteins, selenium polysaccharides, etc.) is predominantly distributed within living organisms. Moreover, organic selenium demonstrates enhanced bioavailability and more efficient assimilation in the human body relative to inorganic selenium. Adequate selenium supplementation is reported to confer benefits such as antioxidant, antitumor, and anti-inflammatory effects [4,5,6]. Despite the status of selenium as a nutritional supplement, its toxicity threshold only slightly exceeds daily bodily requirements [7]. Selenium deficiency is suggested as a primary factor contributing to Keshan disease (KD) [8], while excessive selenium exposure has been associated with conditions such as diabetes and adverse effects on a wide range of neurological functions [9]. The narrow safety margin associated with selenium consumption, along with the lack of universally acknowledged recommended dosages and standardized dietary sources, emphasizes the need for refined guidelines regarding the intake of both organic and inorganic selenium species. This highlights the importance of focusing on experimental studies with individual selenium compounds rather than solely considering overall elemental exposure [10]. Honey, traditionally renowned for its widespread use, is derived from nectar, which serves as a natural repository of selenium. Through enzymatic processes during honey production by bees, the diverse array of selenium compounds found in honey underscores its dual role as a favorable selenium supplement and a valuable sample for analysis. Moreover, honey also possesses antimicrobial, anti-inflammatory, and antioxidant properties [11,12,13]. The comprehensive analysis of the various constituents present in honey is of significant academic interest due to its potential implications for human health.

Selenium, recognized as a significant mineral, is commonly assessed in honey using Inductively Coupled Plasma (ICP) systems and Atomic Absorption Spectroscopy (AAS) for trace element determination [14]. Although AAS maintains advantages in instrumentation cost relative to ICP-based techniques, ICP methods have increasingly replaced AAS in many analytical contexts owing to their superior speed for multi-element analysis, wider elemental scope, and enhanced throughput [15]. Nevertheless, both methods are restricted to evaluating the total selenium content in the sample, lacking precise qualitative and quantitative analysis of individual selenium compounds. While the combination of ICP systems with High-Performance Liquid Chromatography (HPLC) can aid in the analysis of both organic and inorganic selenium in samples, it is important to note that the pretreatment procedure associated with HPLC is characterized by complexity and extended analysis duration [16]. HPLC or Ultra-High-Performance Liquid Chromatography (UHPLC), coupled with Mass Spectrometry (MS), is predominantly chosen for the analysis of different types of amino acids in honey [14]. Although MS coupling offers excellent sensitivity and selectivity, the sample pretreatment process is intricate [17,18]. Gas chromatography–mass spectrometry (GC-MS) can also be proposed for amino acid analysis in honey, providing shorter analysis times than HPLC, but it requires derivatization, consisting of solid-phase extraction, prior to analysis [19,20]. Notably, nuclear magnetic resonance (NMR), such as ^1^HNMR analysis of honey, is rapid and requires minimal sample quantities [21], but the interpretation of proton spectra is often complex, especially in the presence of multiple compounds, leading to spectral overlap and increased analytical challenges [22].

Based on the specified technological limitations in identifying selenium compounds within honey, our research employs reactive EESI-MS methodology. It has been noted that selenite can engage in complexation reactions with *o*-Phenylenediamine (OPD), leading to the creation of 1,3-dihydro-2,1,3-benzoselenadiazole [23,24]. Through the addition of an OPD solution to honey, selenite present therein can transition from an inorganic to an organic state. EESI-MS provides significant advantages in analyzing complex samples, featuring high sensitivity, high selectivity, and rapid response, often eliminating the need for extensive sample pretreatment [25]. Following EESI ionization, diluted honey samples undergo direct mass spectrometric analysis, facilitating real-time detection of selenium compounds. To date, only over 20 Se compounds have been identified explicitly in food samples (including honey) as of now [26,27]. In this study, considering the prevalence and representativeness in food matrices, we adopted sodium selenite (representing inorganic selenium) and selenomethionine (representing organic selenium) as the representatives for analysis. This methodology not only allows for a comprehensive evaluation of selenium content within honey but also enables quantitative analysis of specific selenium species. Such an approach holds promising implications for guiding scientific endeavors in selenium supplementation and refining standards for selenium intake, thereby bolstering advancements in human health and nutritional sciences.

## 2. Results and Discussion

### 2.1. EESI-MS Spectrum

The relative atomic mass of selenium (Se) is 78.96 [28]. Due to the presence of six naturally occurring isotopes [29], careful consideration of selenium isotopes is imperative in analytical procedures. Attention is primarily directed toward the most abundant isotope, ^80^Se, which makes up 49.61% of natural selenium. However, the potential impacts of other isotopes, such as ^78^Se, ^76^Se, ^82^Se, ^77^Se, and ^74^Se, on the analysis of target selenium compounds have also been meticulously evaluated [26].

**Figure 1:**
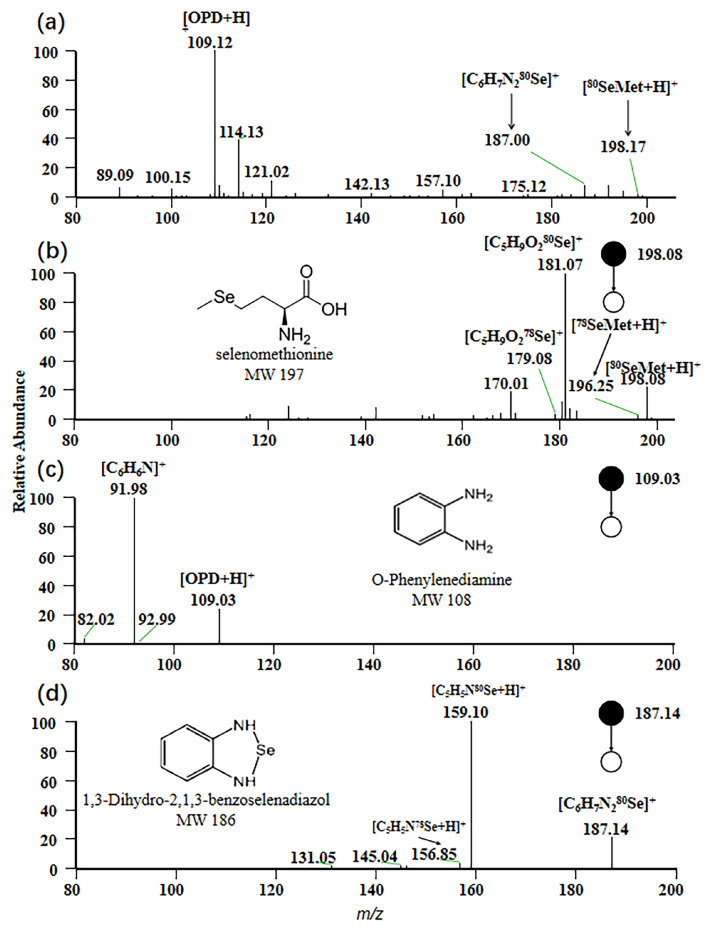
Mass spectrum of selenium compounds. (**a**) MS spectrum of the standard mixed solution; (**b**) MS^2^ spectrum of selenomethionine; (**c**) MS^2^ spectrum of *o*-phenylenediamine; (**d**) MS^2^ spectrum of 1,3-dihydro-2,1,3-benzoselenadiazole.

For EESI-MS, the positive ion mode was selected to obtain a characteristic fragmentation pattern. In this study, the mass spectrum of a standard mixed solution with a concentration of 1 ppm is depicted in Figure 1a. A peak signal at *m*/*z* 198 was observed, corresponding to the adduct ion [^80^SeMet + H]^+^, which represents the protonated form of selenomethionine with a molecular weight of 197 Da. At *m*/*z* 109, a peak signal corresponding to the protonated form [OPD + H]^+^ of OPD under positive ion mode was generated, while the adduct ion [C_6_H_6_N_2_^80^Se + H]^+^ with *m*/*z* 187 was observed, resulting from the protonated form of 1,3-dihydro-2,1,3-benzisoselenazole formed by the reaction of OPD with sodium selenite in the sample.

The MS/MS spectrum of the precursor ion [^80^SeMet + H]^+^ is shown in Figure 1b. A prominent product ion at *m*/*z* 181 is observed, attributed to the loss of NH_3_ from the parent ion [^80^SeMet + H]^+^ during collision-induced dissociation (CID). This fragmentation originates from the signal of [C_5_H_9_O_2_^80^Se]^+^ [30]. Similarly, Figure 1c displays the MS/MS spectrum of the precursor ion [OPD + H] ^+^, with the main product ion peak at *m*/*z* 159. This peak is generated by [C_6_H_6_N]^+^, arising from the loss of NH_3_ from [OPD + H]^+^. The MS/MS spectrum corresponding to the precursor ion [C_6_H_7_N_2_^80^Se]^+^ is presented in Figure 1d. Protonation of 1,3-dihydro-2,1,3-benzisoselenazol in positive ion mode, followed by fragmentation via CID that results in the loss of -CHNH, leads to the formation of the main product ion [C_5_H_5_N^80^Se]^+^ with *m*/*z* 159 [31].

### 2.2. Experimental Optimization

To optimize the experiment, the peak signal intensities of ions primarily generated in a mixed standard solution of 1 mg/L were utilized as indicators. The main product ions included fragment ions with [C_5_H_9_O_2_^80^Se]^+^ (*m*/*z* 181) derived from selenomethionine and [C_5_H_5_N^80^Se]^+^ (*m*/*z* 159) from 1,3-dihydro-2,1,3-benzoselenadiazole. Several parameters in the experimental procedure were adjusted, such as the flow rate of the spray solution, the flow rate of the sample solution, the capillary temperature, and the spray voltage. Other detection parameters were automatically optimized by the LTQ—Tune system. The ion transfer tube was operated at an applied voltage of 35 V, and the lens voltage was set to 110 V.

**Figure 2:**
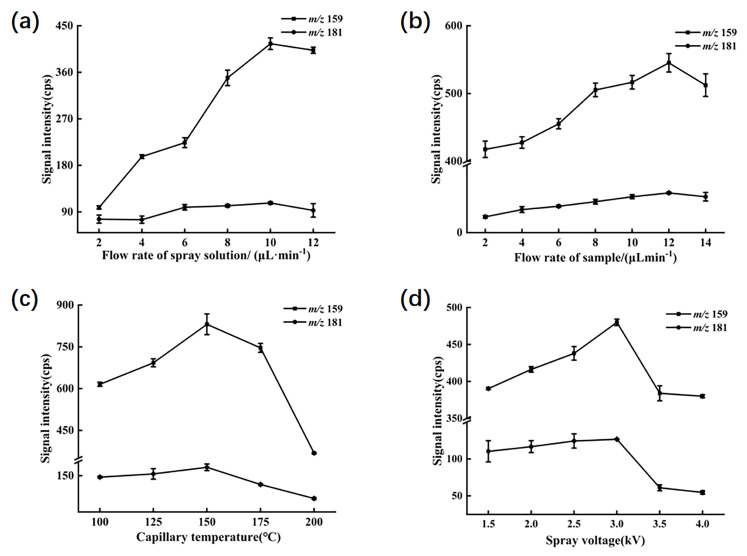
The MS responses of SeMet and sodium selenite to different parameters. (**a**) Ion signal intensities of SeMet and sodium selenite under different flow rates of spray solution. (**b**) Ion signal intensities of SeMet and sodium selenite under different flow rates of sample solution. (**c**) Ion signal intensities of SeMet and sodium selenite at different capillary temperatures. (**d**) Ion signal intensities of SeMet and sodium selenite at different spray voltages.

In the experiment, it was observed in Figure 2a that within the spray solvent flow rate range of 2 to 10 µL/min, the signal intensity for *m*/*z* 159 gradually increased, peaking at 10 µL/min, beyond which the signal weakened. Conversely, the signal at *m*/*z* 181 showed a slight decrease within the 2 to 4 µL/min range, but predominantly exhibited an increasing trend within the 2 to 10 µL/min range, followed by a decline beyond 10 µL/min. This suggests that maintaining a spray solvent flow rate of 10 µL/min yields the highest extraction efficiency for both target compounds; thus, the flow rate was selected for the experiment.

As depicted in Figure 2b, the signal intensities for *m*/*z* 159 and *m*/*z* 181 consistently increased within the sample flow rate range of 2 to 12 µL/min, reaching their peak responses at a flow rate of 12 µL/min. This indicates that a flow rate of 12 µL/min within the range of 2 to 14 µL/min provides optimal conditions for detecting target ions in the sample.

The capillary temperature and spray voltage in EESI play pivotal roles in modulating its ionization and extraction efficiency. Incrementally raising the spray voltage enhances ionization efficiency within the sample solution, although excessive voltage may lead to charge overload in the electrified droplets, potentially causing corona discharge at the spray tip and reducing the analytical sensitivity of the mass spectrometer to target compounds. Meanwhile, the capillary temperature influences the evaporation rate of the solution and the formation of the spray. Moderately augmenting the capillary temperature renders molecules more facile to disengage from the spray and enter the mass spectrometer. Nonetheless, excessive temperatures may engender molecular loss within the solution, precipitating diminution in signal intensity. As illustrated in Figure 2c,d, peak signal intensities for both target ions were maximized at a capillary temperature of 150 °C and a spray voltage range of 3 kV. Consequently, these settings were chosen as optimal for the experiment.

### 2.3. Performance

**Figure 3:**
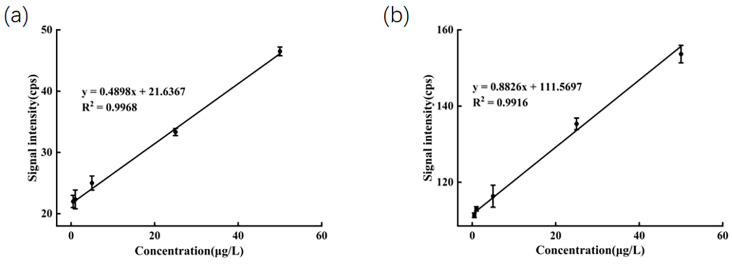
(**a**) Calibration curve of SeMet in diluted honey; (**b**) calibration curve of Na_2_SeO_3_ in diluted honey.

To assess the linearity, LOD, and LOQ of the method, diluted honey solutions spiked with mixed standards at concentrations ranging from 0.5 to 50 µg/L were prepared. The ions *m*/*z* 198 and *m*/*z* 159 were selected as the quantifier ions. After accounting for the blank background, the average signal intensities for these ions were plotted against their corresponding concentrations to construct standard calibration curves. Each sample was measured in triplicate. As shown in Figure 3, a strong linear correlation was observed within the concentration range of 0.5 to 50 µg/L. The determination coefficient (R^2^) was 0.9968 for SeMet and 0.9916 for sodium selenite, respectively. The linear regression equations were formulated as y = 0.4898x + 21.6367 for SeMet and y = 0.8826x + 111.5697 for sodium selenite. LOD and LOQ were calculated based on signal-to-noise ratios of 3:1 and 10:1, respectively. The formulas are as follows:

LOD = 3.3 *s*/*S*

LOQ = 10 *s*/*S*

where *s* is the standard deviation of 10 blank response values, and *S* is the slope of the calibration curve [32]. The findings are shown in detail in Table 1.

The experimental setup involved a full scan time of 100 ms and a collision time of 30 ms in tandem mass spectrometry. The average analysis time per sample was approximately two min, which is significantly faster than the conventional HPLC-MS and GC-MS methods typically used for honey analysis. This method represents a considerable improvement in analytical efficiency. Furthermore, the developed method for analyzing selenium compounds in honey offers several advantages, including shortened analysis time and elimination of intricate sample pretreatment procedures, and has been experimentally validated to exhibit improved sensitivity and reproducibility. This method effectively circumvents complex sample pretreatment procedures and only requires simple dilution to conduct analysis. However, this operation still exhibits a certain gap from the requirements of ideal in situ analysis. Subsequent research will focus on technical optimization and strive to achieve in situ direct analysis of complex samples, so as to further enhance the applicability and analytical efficiency of the method.

### 2.4. Detection of Honey from Different Origins

Analyses were conducted on honey samples from various regions to determine the levels of organic selenium (SeMet) and inorganic selenium (selenite). The samples comprised Guanshengyuan^®^ multifloral honey (Shanghai, China), Fenghe^®^ multifloral honey (Enshi, Hubei, China), and Y. Impression^®^ linden honey (Changbai Mountain, Jilin, China). Before analysis, a hypothesis was formulated regarding a strong correlation between selenium content in honey and the selenium content in the soil of its origin [33]. This is because plants absorb inorganic selenium from soil, which undergoes conversion into organic selenium, subsequently accumulating in honey via pollen and nectar. As indicated in Table 2, Fenghe^®^ multifloral honey (Enshi, Hubei, China) exhibited the highest SeMet and selenite levels at 38.4 µg/kg and 12.2 µg/kg, respectively. This aligns with our expectations, given that the Enshi region in Hubei is known as the “world capital of se.” due to its rich soil selenium content [34,35]. Guanshengyuan^®^ multifloral honey (Shanghai, China) showed a SeMet concentration of 10.4 µg/kg with no detectable selenite, while Y. Impression^®^ linden honey (Changbai Mountain, Jilin, China) had a SeMet concentration of 19.9 µg/kg, also with no detectable selenite. The RSD% of each compound in the three batches of samples was less than 5% (*n* = 3).

To validate the accuracy of the method, recovery experiments were performed using Fenghe^®^ multifloral honey (Enshi, Hubei, China) as a standard sample. Honey samples of Fenghe^®^ multifloral honey (Enshi, Hubei, China) were prepared at three distinct concentrations (70 µg/kg, 140 µg/kg, 280 µg/kg) of spiked selenium (seMet and selenite) for analyzing peak recovery rates, and the formulas used are illustrated below:

Recovery = (C1 − C2)/C0 × 100%

where C1 represents the determined concentration after spiking, C2 is the denotes concentration of the sample, and C0 corresponds to the concentration of the added standard [36].

The results revealed that the average recovery rates varied from 90.6% to 105.7%, with RSDs ranging between 1.73% and 6.03%, indicating the accuracy of the method, as depicted in Table 3.

## 3. Materials and Methods

### 3.1. Materials

Samples comprise Guanshengyuan^®^ Multifloral Honey (Shanghai, China), Fenghe^®^ Multifloral Honey (Enshi, Hubei, China), and Y. Impression^®^ Linden Honey (Changbai Mountain, Jilin, China), meticulously stored at ambient temperature under light-shielded conditions.

### 3.2. Instruments and Reagents

The EESI ion source was custom-developed in the laboratory. The LTQ linear ion trap mass spectrometer (Thermo Fisher Scientific Co., Ltd., San Jose, CA, USA) is equipped with the Xcalibur data processing system. The XPR204S/AC electronic balance is sourced from METTLER TOLEDO (Zurich, Switzerland).

Methanol (chromatographically pure) was purchased from LiChrosolv^®^ solvents (Merck, Darmstadt, Germany). Ultrapure water was prepared by Field-X Ultrapure Water Equipment of Feld Scientific Instrument Co., Ltd. (Beijing, China). *o*-Phenylenediamine (≥98% purity) was purchased from Macklin ^®^ (Shanghai, China). Selenomethionine (≥98% purity) was purchased from J&K Scientific (Beijing, China). Sodium selenite pentahydrate (≥98% purity) was purchased from Aladdin ^®^ (Shanghai, China).

### 3.3. Reactive EESI-MS Analysis

EESI-MS experiments were conducted on an LTQ linear ion trap mass spectrometer. The ion transfer tube was maintained at 150 °C, with an applied voltage of 35 V. Lens voltage was set to 110 V, while the spray voltage was adjusted to 3 kV. The LTQ-Tune system automatically optimized other detection parameters. For tandem mass spectrometry studies, parent ions were isolated with a single mass/charge unit width, and collision-induced dissociation (CID) was performed with 25–35% collision energy, with a collision time of 30 ms. The mass separation width of the parent ions was ±2 Da. The mass spectrometry scan range spanned from *m*/*z* 50 to 500. Thermo Xcalibur Roadmap 2.0 was utilized as the software for mass spectrometry data analysis.

**Figure 4:**
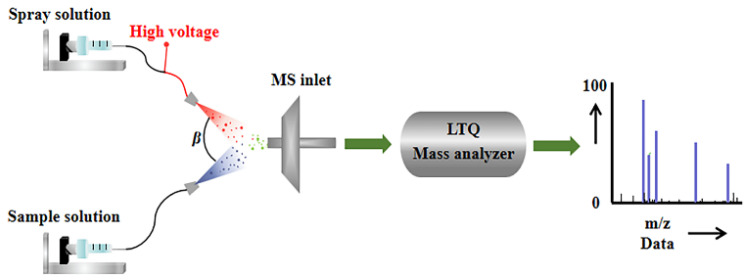
Structure details of EESI-MS.

As shown in Figure 4, in the upper spray channel, methanol was served as the extracting agent, with a flow rate of 10 μL/min. The spray solvent is carried by N_2_ and subjected to a 4 kV voltage to form charged mist-like droplets at the ESI spray tip. In the lower spray channel, sample solution rides high-pressure carrier gas (N_2_) with a flow rate of 12 μL/min to form tiny neutral atomized droplets [37]. Then the droplets formed in these two independent channels collide at the entrance angle (β) of the mass spectrometer, resulting in instantaneous liquid–liquid microextraction. Thus, molecules in the sample are extracted and ionized by the solvent, ready to enter the quadrupole mass filter, where the selected species is ionized [38]. Considering the physicochemical properties unique to honey, prior to conducting EESI experiments, 1 mL of the honey sample was diluted with a methanol–water solution (1:1) to achieve a final volume of 20 mL. During the experimental procedure, 10 mL of the diluted honey sample was supplemented with 20 μL of a 1000 mg/L solution of OPD. This addition aimed to ensure an excess of OPD, facilitating the complete transformation of selenite into its organic species, thereby improving the accuracy and reliability of the detected signals corresponding to the selenite reaction products in the mass spectrometric analysis.

## 4. Conclusions

Prior to the analysis of selenium species in honey via extractive electrospray ionization mass spectrometry (EESI-MS), a preliminary derivatization reaction with an excess of *o*-phenylenediamine (OPD) solution was employed. This strategy enables the simultaneous detection of both organic and inorganic selenium species in honey samples, thereby enhancing the efficiency of selenium compound determination. Specifically, this approach offers several notable advantages: precise quantification of selenium species in food matrices, rapid analytical throughput, minimal sample preparation requirements, and improved detection sensitivity. Moreover, it facilitates comprehensive profiling of selenium compounds in tandem with other matrix components intrinsic to honey samples.

In summary, the proposed methodology provides a streamlined and efficient strategy for assessing selenium compounds in honey and evaluating its overall quality, with sodium selenite and selenomethionine utilized as representatives for the quantification of inorganic selenium and organic selenium, respectively. However, it should be noted that there are various selenium compounds existing in the honey matrix. Thus, this method may potentially have limitations in the accurate quantification of other selenium compounds. Additionally, certain limitations exist in this study. To ensure the reproducibility and scientific rigor of the experimental results, further verification experiments and expanded investigations with larger sample sizes are warranted.

## Figures and Tables

**Table 1:** Characterization of two selenium compound forms with EESI-MS.

Form	Quantitative Ions (*m*/*z*)	Quantitative Limits (µg/L)	Liner Equation	R^2^	^※^ LOD (µg/kg)	^※^ LOQ (µg/kg)
SeMet	181	0.5–50	y = 0.4898x + 21.6367	0.9968	2.94	9.52
Selenite	159	0.5–50	y = 0.8826x + 111.5697	0.9916	5.18	17.4

^※^ LOD and LOQ refer to the undiluted sample system.

**Table 2:** Contents of two forms of selenium compounds in honey samples.

Form	Sample	Determined Concentration (µg/kg)	RSD (%) *n* = 3
	Guanshengyuan^®^ multifloral honey (Shanghai, China)	10.4	4.54
SeMet	Fenghe^®^ multifloral honey (Enshi, Hubei, China)	38.4	4.34
	Impression^®^ linden honey (Changbai Mountain, Jilin, China)	19.9	2.59
	Guanshengyuan^®^ multifloral honey (Shanghai, China)	/	/
Selenite	Fenghe^®^ multifloral honey (Enshi, Hubei, China)	12.2	1.36
	Impression^®^ linden honey (Changbai Mountain, Jilin, China)	/	/

**Table 3:** Results of spike recovery experiments for two selenium compounds in honey samples.

Form	Sample Concentration (µg/kg)	Added Concentration (µg/kg)	Determined Concentration (µg/kg)	Recovery (%)	RSD (%) *n* = 3
		70	105.7	96.1	6.03
SeMet	38.4	140	172.3	95.7	5.52
		280	334.3	105.7	1.73
		70	80.8	98.1	4.83
Selenite	12.2	140	139.0	90.6	4.63
		280	276.5	94.4	2.78

## Data Availability

The original contributions presented in this study are included in the article. Further inquiries can be directed to the corresponding author.

## Abbreviations

The following abbreviations are used in this manuscript:

EESI-MS	Extractive electrospray ionization mass spectrometry
MS	Mass Spectrometry
GC-MS	Gas chromatography–mass spectrometry
ICP	Inductively Coupled Plasma
ASS	Atomic Absorption Spectroscopy
HPLC	High-Performance Liquid Chromatography
UHPLC	Ultra-High-Performance Liquid Chromatography
NMR	Nuclear magnetic resonance
LOD	The limits of detection
LOQ	The limits of quantification
CID	Collision-induced dissociation
OPD	*o*-Phenylenediamine
SeMet	Selenomethionine
Se	Selenium
KD	Keshan disease
